# Role of EGFR gene polymorphisms in oral squamous cell carcinoma patients of Southeast Iran: A case-control study

**DOI:** 10.22088/cjim.11.4.391

**Published:** 2020

**Authors:** Shirin Saravani, Negin Parsamanesh, Ebrahim Miri-Moghaddam

**Affiliations:** 1 Oral and Dental Disease Research Center & Department of Oral and Maxillofacial Pathology, Faculty of Dentistry, Zahedan University of Medical Sciences, Zahedan, Iran; 2 Students Scientific Research Center, Faculty of Medicine, Birjand University of Medical Sciences, Birjand, Iran; 3 Cardiovascular Disease Research Center & Department of Molecular Medicine, Faculty of Medicine, Birjand University of Medical Sciences, Birjand, Iran

**Keywords:** Oral squamous cell carcinoma, Epidermal growth factor receptor, Polymorphisms

## Abstract

**Background:**

The decisive etiology of oral squamous cell carcinoma (OSCC) is still ambiguous, but we recognize the contribution of genetic aberration and environmental agents due to OSCC initiation. In the current study, we elucidate the potential impact of EGFR gene polymorphisms on the risk of OSCC in Southeast Iran.

**Methods:**

Forty-eight OSCC patients along with 100 healthy volunteers were included. Three polymorphisms of the EGFR gene (rs2227983, rs2293347 and rs2227984) were genotype by Tetra-ARMS PCR. Data were analyzed with a chi-square test, and p<0.05 was considered significant.

**Results:**

In rs2227983, the frequency of AG and GG genotypes were 62.5%, 37.5% in cases and 42%, 57% in the control group (P=0.02, OR=2.3) and also A allele frequency was 31.3% in the case and 22% in control (P=0.08, OR=0.62). AG + AA genotype frequency was 62.5% and 43% in case and control, respectively (p=0.03, OR=2.2). In rs2227984 and rs2293347, no statistical differences showed in the distribution of genotypes between the case and control group. Also the majority of the OSCC belonged to grade I (43.8%).

**Conclusion:**

The present investigation indicated that rs2227983 polymorphism might contribute to OSCC susceptibility in Iran's southeast population. Although, with the inconsistent interpretation mentioned due to the various geographical residencies and populations, more studies of significant populations are suggested to validate our findings.

Head and neck neoplasm is the sixth most widespread epithelial cancer worldwide (1). Oral squamous cell carcinoma (OSCC) is represented in more than 90% of oral cavity malignancies (2). OSCC affected different oral anatomical positions including tongue, lip, oropharynx, mouth floor, hard palate, gingiva and buccal mucosa (3). In America, its incidence rate is 2% to 4% of all cancers every year and approximately responsible for 8,000 mortality annually (2). Also, in Iran as an area located in the Middle East, a study manifested similar epidemiology of southern Asian countries ,about 20-36 cases in 100,000 people (4). Genetic susceptibility and oral habits collaboration including alcohol consumption and cigarette smoke have an excessive effect on carcinogenesis (5). However, the definitive tumorgenesis pathway is ambiguous yet, regarding the molecular defect accumulation in a biological process such as tumor suppressor genes, proto-oncogenes and cell signaling may be due to the oral carcinogenesis in numerous stages (1). The receptor tyrosine kinases (RTK) are cell surface receptor family organized from multiple subgroups including epidermal growth factor receptor (EGFR), insulin-like growth factor receptor (IGFR), fibroblast growth factor receptor (FGFR) and vascular endothelial growth factor receptor (VEGFR) (6). 

RTKs are operated as receptors for hormones, growth factors, cytokines and extracellular signaling molecules (7). Moreover, these receptors could regulate main biological processes such as cell proliferation, cell transformation, differentiation, survival and migration in normal and a tumor cell (8). 

The EGFR gene is placed on chromosome 7p and generates high-affinity glycoprotein such as transforming growth factor alpha (TGFα**)** and epidermal growth factor (EGF) (9). This receptor participates in signal transduction pathways including DNA maintenance, cancer cell survival and invasion (10). EGFR overexpression occurred in several carcinogenesis, and also numerous reports have shown the relation between the poor prognosis and decreased overall survival (9). In addition, EGF-like growth factors have been detected in most human tumors that can attach ErbB receptors and mediate pro-angiogenic factor secretion (11). Evidence indicates that EGFR upregulation has a significante role in several carcinoma cell progression and assumed over expression of ErbB receptor is involved in several solid neoplasms (12). 

Furthermore, both the in-vitro and in vivo studies have displayed the potential impact of these proteins in cell transformation (13). Increasing EGFR expression in oral malignancies is closely correlated with disturbance behavior, decrease apoptosis and has an effective role in cancer invasion (14). However, there are controversial reports of EGFR act in tumorgenesis, Bossi et al. indicated that nuclear EGFR variation, EGFR phosphorylation and TGF-α level can be as predictive factors in patients with head and neck squamous cell carcinoma treated by EGFR inhibitor drugs (15). Bandres et al. in 2007 demonstrated that EGFR genotypes could be helpful markers in metastatic or recurrent OSCC patient’s survival outcomes. Furthermore, their study suggested that EGFR polymorphisms can be beneficial in EGFR-targeted antibody therapies (16). Elie et al. revealed a lack of association between EGFR expression and overall and progression-free survival in ovarian cancer patients (17).

Molecular epidemiologic studies in 2006 showed that EGFR gene variations might be related to alteration in cellular biological activities (12). Nevertheless, the impact of functional and actual frequency EGFR genotypes of Sistan-Baluchestan province OSCC patient population has not been reported yet, here we probably study the correlation between single nucleotide polymorphisms (rs2227983, rs2227984 and rs2293347) of EGFR polymorphisms and OSCC susceptibility to elucidate the precise role in the sample Iranian population.

## Methods

**Study population** **:** In this case-control study, forty-eight OSCC patients and 100 healthy subjects were studied. All of the OSCC patients were histologically confirmed by two maxillofacial pathologists that referred to the Dentistry Faculty of Zahedan University of Medical Sciences in 2017. All histopathological slides of OSCC samples were classified into three grades, including well-differentiated (grade I), moderately differentiated (grade II) and poorly differentiated (grade III) by maxillofacial pathologists (18).

Case and control subjects were matched by age, gender and ethnicity. This research was received, accepted, and coded by the ethics committee of this university (IR.ZAUMS.REC.1394.379).

**DNA isolation** **and polymorphisms genotyping:** Genomic DNA was isolated from the paraffin-embedded tissue blocks of OSCC patients by standard extraction procure (19), while blood leukocytes were used for the normal group. 

The EGFR single nucleotide polymorphisms rs2227983(R497K), rs2227984 (T584T) and rs2293347 (D994D**)** were genotyped using tetra-ARMS PCR techniques. The primer sequences and product size have been summarized in table 1. PCR reaction contained genomic DNA (50 ng), 1µl each of primer and 10 µl of Taq DNA polymerase master mix red (Amplicon, Denmark ) and added water to reach the final volume of 20 µl. PCR was done with temperature profile as follows: initial denaturation step (95 °C for 5 min), followed by 30 cycles of denaturation (95°C for 1 min), annealing (69°C for 1 min for rs2227984, 62°C for rs2293347 and rs2227983) and extension step (72°C for 1 min), and final extension (72°C for 5 min). The DNA fragments were analyzed by electrophoresis on 2 % agarose gel and safe stained (Cinna Gen, Iran), then visualized with UV light (cleaver, UK).

**Statistical analysis** **:** Statistical analysis was performed by *SPSS 16.0 *software package (SPSS Inc, Chicago, IL). The chi-square test was used to compare quantitative variables between the two groups. The odds ratios (ORs) and 95% confidence intervals (CIs) for overall OSCC were estimated, seeking the probable correlation between EGFR polymorphisms and OSCC susceptibility. A p-value < 0.05 was considered remarkable.

**Table 1:** Primer sequences and product size were used for EGFR genotyping by tetra-ARMS methods

**SNP**	**Primers sequence**	**Product size (bp)**
rs2227983	F-outer: 5′-CAC TCT GTC TCC GCA GAG GCC ACA GG-′3 R-outer:5′-GGA GCC TTA TTT TTG ATC AAC GCA AGG GG-′3 F-inner: 5′-CTG CTG GGG CCC GGA GCC AAG-′3 R-inner: 5′-TGA CAT TCC GGC AAG ACG CAG TAC T-′3	Outers: 200 Inner G: 143 Inner A: 106
rs2293347	F-outer: 5′-TTG TTC AAA TGA GTA GAC AGC TTG AGA-′3 R-outer: 5′-TAA CAA AAT TGG CAA ACA CAC AGG C-′3 F-inner: 5′-CAT CAG GGC ACG GTA GAA GTT GTA A-′3 R-inner: 5′-AAG AAT GCA TTT GCC AAG TCC TAC ATA C-′3	Outers: 339 Inner G: 215 Inner A: 117
rs 2227984	F-outer: 5′-TTA ACC ACC AAT CCA ACA TCC AGA C-′3 R-outer: 5′-CAG GAC AGA GGA CAG TCA GAA ATG C-′3 F-inner: 5′-CTC TTT CAC TTC CTA CAG ATG CGC T-′3 R-inner: 5′-GAC AGC CTT CAA GAC CTG GCT CT-′3	Outers: 294 Inner A: 184 Inner T: 159

## Results

In the current research, three single nucleotide polymorphisms assessed in the EGFR gene in 48 OSCC patients include 17 (35.42%) males, 31 (64.58%) females and 100 control individuals, 57 females and 43 males. The mean age of the case and control groups were 58±13 and 55±10 years, respectively. As shown in table 2, in rs2227983, the frequency of heterozygous AG (P=0.02, OR=2.3) and AG+AA genotype (P=0.03, OR=2.2) were significantly higher in cases than the control group. still, the difference of A allele frequency was not statistically significant (P=0.08, OR=0.62). In rs2293347 and rs2227984, no statistical difference was shown in the distribution of genotypes and alleles. The majority of the OSCC belonged to grade I (43.75%), and 19 and 8 cases belonged to grades II and III, respectively. However, no significant relation was detected between OSCC grade and EGFR genotypes (table 3). Tumors located at mandibular gingiva were (19 cases, 39.58%), in the buccal mucosa (14 cases, 29.17%), maxillary gingiva (7 cases, 14.58%), tongue (5 cases, 10.42%), and in the ventral surface of the tongue (3 cases, 6.25%).

**Table 2:** EGFR genotypic and allelic frequency in OSCC cases (n = 48) and healthy (n = 100) objects

**Gene**	**Accession number **	**SNP**	**R/MA allele**	**Genotype**	**Case N (%)**	**Control N (%)**	**P-value**	**OR**
EGFR	rs2227983	R476K	A/G	GG	18 (37.50)	57 (57.00)	Ref:1	
				AG	30 (62.50)	42 (42.00)	0.02*	2.30
				AA	0 (0.00)	1 (1.00)	0.76	1.01
				AG+AA	30 (62.50)	43 (43.00)	0.03*	2.20
				Allele G	66 (68.75)	156 (78.00)	Ref:1	
				Allele A	30 (31.25)	44 (22.00)	0.08	0.62
	rs2227984	T584T	A/T	TT	15 (31.25)	39 (39.00)	Ref:1	
				AT	23 (47.91)	50 (50.00)	0.40	0.83
				AA	10 (20.83)	11 (11.00)	0.08	0.42
				AA+AT	33 (68.75)	61 (61.00)	0.23	0.71
				Allele T	53 (55.20)	128 (64.00)	Ref:1	
				Allele A	43 (44.80)	72 (36.00)	0.09	0.69
	rs2293347	D994D	A/G	GG	30 (62.50)	56 (56.00)	Ref:1	
				AG	15 (31.25)	34 (34.00)	0.37	1.21
				AA	3 (6.25)	10 (10.00)	0.30	1.78
				AG+AA	18 (37.50)	44 (44.00)	0.28	1.31
				Allele G	75 (78.12)	146 (73.00)	Ref:1	
				Allele A	21 (21.87)	54 (27.00)	0.21	1.32

*; remarkable values in bold

**Table 3:** Distribution of EGFR variation in OSCC cases according to histopathological grades

**SNP**	**Genotype**	**Grade I** **N (%)**	**Grade II** **N (%)**	**Grade III ** **N (%)**	**P-value**
rs2227983	GG	5 (23.80)	9 (47.36)	4 (50.00)	0.22
	AG	16 (76.20)	10 (52.63)	4 (50.00)	
	AA	0 (0.00)	0 (0.00)	0 (0.00)	
rs2227984	TT	14 (66.66)	12 (63.15)	4 (50.00)	0.98
	AT	6 (28.57)	6 (31.57)	3 (37.50)	
	AA	1 (4.76)	1 (5.26)	1 (12.50)	
rs2293347	GG	7 (33.33)	5 (26.31)	3 (37.50)	0.93
	AG	9 (42.85)	10 (52.63)	4 (50.00)	
	AA	5 (23.80)	4 (21.05)	1 (12.50)	

## Discussion

OSCC is a multifactorial disease represented in about 90% of oral cancer worldwide (1). Most studies demonstrated that hereditary and environmental factors contributions are the major etiology in cancer development (20). Numerous human malignancies displayed EGFR expression, which is linked to a neoplasm progression and tumor grades along with cancer poor prognosis (21). EGFR is a transmembrane tyrosine kinase receptor that displays a maintenance act in signal transduction pathways including DNA repair, cell proliferation, tumor survival and invasion. The majority of patients with head and neck squamous cell carcinoma treated with a cetuximab-based therapy have been shown, EGFR variation could be a useful biomarker for less skin toxicity and poor prognosis (10, 22). In the current study, we evaluated the potential impact of three genotypes of the EGFR gene; rs2227983, rs2227984, and rs2293347 in OSCC patients of the southeast of Iran. 

- In rs2227983, with a G→A substitution leading to an Arginine (Arg) →Lysine (Lys) change in codon 497. The results showed that the heterozygous AG variation was common in patients than the control individuals. Also, results indicated that the presence of A allele at rs2227983 polymorphic site (AG + AA) of the EGFR gene is related to OSCC susceptibility and this genotype is in association with OSCC predisposition. With regard the allelic distribution of G and A in rs2227983, the respective frequencies were not remarkably diverse from the ratios observed in the control healthy (68% vs 78% and 31 vs 22% respectively). In the USA population, the G and A allele frequencies were observed as 67% and 33 %, respectively, in Southern Asia, showed a similar frequency (G=0.65%, A=0.35) (23). Moriai et al. reported that the variant A has more reduced tyrosine kinase activity than G allele and it can lead to reductions in ligand binding, growth stimulation and induction of proto-oncogenes MYC, FOS and JUN (22). Some evidence demonstrated that the R497K-Lys genotype has not been involved in cancer predisposition and displayed the correlation with the improved clinical outcome in several tumors (24, 25). This polymorphism was studied as a potential predictor in overall survival in HNSCC patients treated with cetuximab. The patients have at least one K-allele that showed shorter overall survival and median survival was 6.7 months compared to 13.3 months in the patients homozygous for the R-allele (26). Stoehlmacher-Williams et al. in 2012 showed that the rs2227983 variation could be a promising prognostic factor for EGFR chemotherapy patients with advanced cancer of the head and neck (26). Su et al. in 2014 indicated that EGFR R521K G>A (rs2227983) genotypes could be critical predictor markers in patients with advanced primary pharyngolaryngeal squamous cell carcinoma treated with cancer drugs concurrently (27).

- In rs2293347, with an A→G substitution leading to an aspartate (ASP) change in codon 994 to the same amino acid in the coding region of exon 25. This synonymous SNP may not involve the biological activity of the protein itself (28). However, it can be influenced by mRNA stability, alternative splicing and translational kinetics and terminated to change of protein quantity, construction and activity (29). The results showed that no statistical distribution was found in frequencies of all genotypes. The study demonstrated that G and A's allelic frequencies in rs2293347 were 78 % and 22% in the case group. These data did not show significant difference from the ratios observed in control group (73 and 27, respectively). The G and A allele distributions in the American population were 85% and 15%, respectively. Moreover, in Southern Asia displays similar distribution (G=77%, A=23%). Ma et al. in 2009 reported that EGFR variation in rs2293347 (D994D) was related to the clinical outcome of Gefitinib treatment in advanced non-small-cell lung cancer (NSCLC) patients, the response rate of GG genotype patients was almost double with that of other genotypes (71.2% versus 37.5%,). Therefore, it may be of functional relevance (30). 

-In rs2227984, with a T→A synonymous substitution leading to threonine (Thr) shift in codon 584 that means replacing a codon with another codon of the same amino acid (31). This study showed that the AA genotype frequency was 20% vs 11% in control groups. Moreover, T and A's allelic distributions were 55% vs. 64% and 45% vs. 36% in case and control groups, respectively. T and A allele frequencies were shown to be 60% and 40 % in the USA population, respectively. The T and A allele spread of these genotypes in the Southern Asia was 56% and 44%, respectively (32). In line with us, Zhang et al. in 2013 study 7 EGFR gene exons in gastric cancer of the Chinese population and could not find any relation between rs2227984 and rs2293347 gastric cancer risk (9). Some evidence reported that different EGFR SNPs, such as rs2293347 and rs2227983, were involved in tumor biological behavior, including tumor metastasis, progression, and could be affected tumorigenesis (33). Some studies indicated that EGFR tyrosine kinase (TK) inhibitor such as gefitinib, was useful for non-small cell lung cancer therapy in Japan in clinical trial phase II and III. In a study, results showed that EGFR polymorphism at exon 25 sites probably are associated with NSCLC progression (34). The meta-analysis and systematic meta-analysis revealed that the EGFR R521K variation is not related to cancer risk, regarding various anticancer therapies may require further studies (35). EGFR plays an essential act in tumorigenesis because of its receptor for several varieties of ligands involved in betacellulin, TGF-𝛼, EGF and heparin-binding EGF-like growth factor (HB-EGF). The binding of ligand ignition in tumor cell function comprises antiapoptosis, proliferation and invasion through triggers of PI3K/Akt/mTOR, JAK/STAT and MAPK pathways activation (36, 37). In this regard, the facility that several genetic variations in the EGFR gene are a controversial hypothesis may apply to engage EGFR-targeted therapies to cancer patients.

In conclusion this study indicated that the EGFR G>A (rs2227983) polymorphism was the promising predictor factor in OSCC patients in the southeast population of Iran. However, our data can not yet definitely emphasize the role of EGFR gene variations. It seems that this receptor participates in essential signaling pathways of cell cycles and then may be used as a biomarker in response to chemotherapy. Although, with the inconsistent interpretation mentioned due to the various geographical residencies, different populations and ethical diversities, more studies in major populations are suggested to be performed to validate our findings.
